# Promotion of *Bamboo Mosaic Virus* Accumulation in *Nicotiana benthamiana* by 5′→3′ Exonuclease NbXRN4

**DOI:** 10.3389/fmicb.2015.01508

**Published:** 2016-01-06

**Authors:** Cheng-Cheng Lee, Tzu-Ling Lin, Jhe-Wei Lin, Yu-Tsung Han, Yu-Ting Huang, Yau-Heiu Hsu, Menghsiao Meng

**Affiliations:** ^1^Graduate Institute of Biotechnology, National Chung Hsing UniversityTaichung, Taiwan; ^2^Division of Medicine Centre for Nephrology, University College LondonLondon, UK

**Keywords:** *Bamboo mosaic virus*, potexvirus, RNA replication, replicase, replication protein, host factors, exonuclease, XRN4

## Abstract

*Bamboo mosaic virus* (BaMV) has a 6.4-kb (+) sense RNA genome with a 5′ cap and a 3′ poly(A) tail. ORF1 of this potexvirus encodes a 155-kDa replication protein responsible for the viral RNA replication/transcription and 5′ cap formation. To learn more about the replication complex of BaMV, a protein preparation enriched in the 155-kDa replication protein was obtained from *Nicotiana benthamiana* by a protocol involving agroinfiltration and immunoprecipitation. Subsequent analysis by SDS-PAGE and mass spectrometry identified a handful of host proteins that may participate in the viral replication. Among them, the cytoplasmic exoribonuclease NbXRN4 particularly caught our attention. NbXRN4 has been shown to have an antiviral activity against *Tomato bushy stunt virus* and *Tomato mosaic virus*. In *Arabidopsis*, the enzyme could reduce RNAi- and miRNA-mediated RNA decay. This study found that downregulation of NbXRN4 greatly decreased BaMV accumulation, while overexpression of NbXRN4 resulted in an opposite effect. Mutations at the catalytically essential residues abolished the function of NbXRN4 in the increase of BaMV accumulation. Nonetheless, NbXRN4 was still able to promote BaMV accumulation in the presence of the RNA silencing suppressor P19. In summary, the replication efficiency of BaMV may be improved by the exoribonuclease activity of NbXRN4.

## Introduction

Replication proteins of positive-stranded RNA viruses form ribonucleoprotein complexes in association with membranes derived from a variety of cytoplasmic organelles (Novoa et al., 2005; Miller and Krijnse-Locker, 2008). The membrane structure may be modified or remodeled dynamically during the RNA replication process. For example, the replication complex of *Hepatitis C virus* resides in spherules derived from endoplasmic reticulum (Quinkert et al., 2005). *Brome mosaic virus* (BMV) and *Red clover necrotic mosaic virus* (RCNMV) also form the viral replication complexes by virtue of the endoplasmic reticulum membranes (Noueiry and Ahlquist, 2003; Turner et al., 2004). *Flock house virus* replicates on the mitochondria membrane (Miller et al., 2001), while *Tomato bushy stunt virus* (TBSV) replicates on the surfaces of peroxisomal membrane (McCartney et al., 2005). Formation of such membrane-derived microenvironments benefits viruses to escape from proteolysis and RNA hydrolysis imposed by the host defense systems.

*Bamboo mosaic virus* (BaMV) has a positive-sense single-stranded RNA genome of approximately 6.4 kb in length, with a 5′ cap structure and a 3′ poly(A) tail. The genome contains five open reading frames (ORFs) plus a 5′ untranslated region (UTR) of 94 nt and a 3′ UTR of 140 nt [without counting the poly(A) tail] (Lin et al., 1994). ORF1 of the virus encodes a ∼155-kDa replication protein consisting of an N-terminal AdoMet-dependent guanylyltransferase (Li et al., 2001a), an RNA 5′-triphosphatase/NTPase (Li et al., 2001b), and a C-terminal RNA-dependent RNA polymerase (RdRp) (Li et al., 1998). ORF2-4, referred as triple gene block, encode three movement proteins necessary for the virus movement in plants (Lin et al., 2004, 2006), and ORF5 directs the synthesis of the viral coat protein (CP). The RNA 5′-triphosphatase/NTPase domain has a strong affinity to the viral CP and this interaction is critical for BaMV to move in plants (Lee et al., 2011). Occasionally, an 836-nt satellite RNA (satBaMV) was found to associate with BaMV infection (Lin and Hsu, 1994). The genome of satBaMV contains a 20-kDa polypeptide-encoding ORF, flanked by a 5′ UTR of 159 nt and a 3′ UTR of 129 nt. The replication of satBaMV is absolutely dependent on BaMV.

Viruses need to hijack host factors to proceed with the infection process, including entry, replication, trafficking, virion assembly, and release from the infected cells. On the other hand, hosts may dispatch proteins to interrupt the viral life cycle. To elucidate the interplay between plant viruses and their hosts, genome-wide screens using *Saccharomyces cerevisiae* as a surrogate host have identified diverse cellular factors capable of affecting the accumulations of BMV (Kushner et al., 2003) and TBSV (Panavas et al., 2005). In other cases, biochemical methods of using the immunopurified replication complex, followed by mass spectrometry, were exploited to find important host components for the replication of TBSV (Serva and Nagy, 2006) and RCNMV (Mine et al., 2010). As for BaMV, UV-induced crosslinking using the radiolabeled 3′ UTR of BaMV as a probe has identified several 3′ UTR-interacting proteins, including chloroplast phosphoglycerate kinase (PGK), cytosolic glyceraldehyde 3-phosphate dehydrogenase (GAPDH), and heat shock protein 90 homolog (NbHsp90). PGK promotes BaMV accumulation presumably by facilitating BaMV targeting to chloroplasts (Lin et al., 2007; Cheng et al., 2013a). GAPDH reduces BaMV accumulation principally by inhibiting the synthesis of the viral negative-strand RNA (Prasanth et al., 2011). NbHsp90, which also interacts with BaMV replication protein, selectively enhances the replication initiation of BaMV but not satBaMV (Huang et al., 2012). Yeast two-hybrid screen hunted out an uncharacterized AdoMet-dependent methyltransferase (PNbMTS) from *N. benthamiana* cDNA library by using BaMV RdRp as bait (Cheng et al., 2009). PNbMTS is a suppressor for BaMV replication. The technique of cDNA-amplified fragment length polymorphism (cDNA-AFLP) has been adopted to analyze the transcript profiling of *N. benthamiana* upon BaMV infection and successfully identified 49 up-regulated genes and 41 down-regulated genes (Cheng et al., 2010). Of those factors, a glutathione transferase (NbGSTU4) promotes BaMV accumulation presumably by providing a more suitable redox environment for BaMV replication (Chen et al., 2013). A serine/threonine kinase-like protein (NbSTKL) is involved in the cell-to-cell movement of BaMV (Cheng et al., 2013b), while a putative Rab-GTPase activation protein (NbRabGAP1) is important for the viral intercellular movement (Huang et al., 2013).

Limited expression of BaMV replication protein in plants has been a bottleneck toward understanding the viral replication complex. A protocol involving agroinfiltration and immunoprecipitation was established in this study to isolate the BaMV replication protein-enriched fraction, from which a handful of *N. benthamiana* proteins were selectively identified by mass spectrometry. Screen based on the expression of green fluorescent protein (GFP) by *GFP*-carrying BaMV in the selected protein-downregulated plants suggested several potential host factors, including cytoplasmic 5′→3′ exoribonuclease (NbXRN4), *S*-adenosylmethionine synthetase, a ripening-related protein, a respiratory burst oxidase homolog, a MAP kinase phosphatase-like protein, and NADP^+^-dependent isocitrate dehydrogenase. The involvement of NbXRN4 in BaMV accumulation was characterized in detail in this study.

## Materials and Methods

### Plasmids

A pEpyon-based binary plasmid, pERep, was constructed previously in an attempt to overexpress BaMV replication protein, fused with a hemagglutinin (HA) tag at the C terminus, in *N. benthamiana* (Lee et al., 2011). pKSF4, a pKn-based binary plasmid, was also created at that time to produce the SF4 variant of satBaMV. For gene-silencing experiments, each of the selected cDNA fragments of the target genes was inserted into the cloning site of pTRV2 (Ratcliff et al., 2001) via restriction sites *Eco*RI and *Xho*I. The pTRV2 derivatives that carry luciferase gene (pTRV2-Luc) and phytoene desaturase gene (pTRV2-PDS) were used as the negative and positive controls in the silencing experiments, respectively. pCBG is a BaMV infectious clone, in which an engineered cDNA copy of BaMV is positioned downstream of the CaMV 35S promoter (Lin et al., 2004). This clone contains a GFP-expression cassette in the BaMV genome so that the expression of GFP could be used as an index for BaMV accumulation. The full-length cDNA of NbXRN4 was amplified from a cDNA library of 6-week-old *N. benthamiana* by PCR using primers (5′-GGGATGGGAGTACCAGCATTTTATA-3′ and 5′-ATGCGAGCTCTTATTGATGTGTTCCTGTTTCTT-3′) and inserted into the transient protein expression vector pBI221 by using *Sma*I and *Sac*I sites. This construct, pBI-XRN4, was used to overexpress NbXRN4 in protoplasts of *N. benthamiana*. Mutagenesis to substitute alanine for Asp55 and Glu206 of NbXRN4 on pBI-XRN4 was performed according to the protocol of the QuikChange site-directed mutagenesis kit (Stratagene). The pairs of divergent primers 5′-GCCATGAATGGTATCATTCACCCT-3′/ 5′-CAAGTACATGTTATCAAATTCCAT-3′ and 5′-GCTGGAGTGGCTCCTAGAGCT-3′/ 5′-AATAGCCATATAGAGGAGTTTTCTTG-3′ were used for D55A and E206A mutations, respectively.

### *Agrobacterium* Infiltration

pERep and pKSF4 were co-infiltrated into leaves of *N. benthamiana* to produce BaMV replication protein. *Agrobacterium tumefaciens* C58C1 strain that harbors each of the binary plasmids was cultivated in LB medium supplemented with kanamycin at 28°C, 200 rpm, for 2 days. The harvested cells after centrifugation were suspended in buffer that contained 10 mM MES [pH 5.5] and 10 mM MgCl_2_ to an OD_600_ of 0.5. Equal volumes of the two cells were mixed and infiltrated into the undersides of leaves of 4-week-old *N. benthamiana*. In gene silencing experiments, *A*. *tumefaciens* C58C1 that carry pTRV1 or pTRV2 derivative was grown, and the mixed cells at 1:1 ratio was used in the infiltration as the protocol described above.

### Virus Inoculation

In general, the effect of gene silencing started to appear within 4–5 weeks after agroinfiltration as evidenced by the emergence of white spotted regions on leaves of plants that had received pTRV1 and pTRV2-PDS. At the time, 0.5 μg virion of the indicated virus was mechanically inoculated into the corresponding leaves of the other silencing plants. The virus-infected leaves were harvested for protein and RNA analysis 4 days post-inoculation. The expression of GFP in leaves by *GFP*-carrying BaMV was also recorded with the Fujifilm LAS-4000 imager.

### Protoplast Transfection

Protoplasts were prepared from 5-week-old *N. benthamiana* leaves according to the protocol of Sheen (2001) with slight modifications. Polyethylene glycol (PEG) 4000-mediated transfection was used to introduce the indicated plasmids into 1 × 10^5^ protoplasts according to the protocol described previously (Cheng et al., 2009). The transfected protoplasts were cultivated at room temperature in growth buffer (0.55 M mannitol-MES [pH 5.7], 1 μM CuSO_4_, 1 μM KI, 1 mM MgSO_4_, 0.2 mM K_2_HPO_4_, 1 mMKNO_3_, 10 mM CaCl_2_, and 30 μg/ml cefatoxime) for the indicated periods of time under a constant light.

### Preparation of the BaMV Replication Protein-Enriched Fraction

Approximately 10 g leaves co-infiltrated with *A. tumefaciens* carrying pERep and pKSF4 were harvested on day 2 after infiltration and homogenized in 20 μl cold extraction buffer, which contained 50 mM Tris [pH 8.0], 120 mM KCl, 15 mM MgCl_2_, 0.1% (v/v) β-mercaptoethanol, 20% (v/v) glycerol, and 0.1 mM phenylmethanesulphonyl fluoride, with a handheld polytron homogenizer (Kinematica). After removal of the debris with cloth filters, the crude extract was centrifuged at 500 × g for 10 min. The supernatant was centrifuged again at 30,000 × g for 45 min. After wash once with extraction buffer, the pellet (P_30_) was thoroughly suspended in 1.5 ml TSG buffer (50 mM Tris [pH 8.0], 0.3% (v/v) Sarkosyl, 2.5% (v/v) glycerol, 10 mM NaCl, and 1X complete EDTA-free protease inhibitor cocktail) and briefly disintegrated with a sonication probe (Misonix Sonicators-Microson XL-2000, BEK Ultrasonics, USA). The sample was centrifuged again at 30,000 × *g* for 45 min and the supernatant (S_30Sark_) was collected. To 1 ml S_30Sark_, 50 μl anti-HA agarose beads (Abcam, USA) was added, and the mixture was gently shaken overnight. The beads were washed with five 1-ml volumes of TSG buffer and saved at - 80°C. The sample collected by the beads is regarded as the BaMV replication protein-enriched fraction. All the steps of the procedure were performed at 4°C.

### Host Factor Identification by Mass Spectrometry

To the anti-HA agarose precipitate, 30 μl protein sample buffer (0.2 M Tris [pH 6.8], 10% glycerol, 4 mM DTT, 4% SDS, 0.025% bromophenol blue, and 1.6 M urea) was added. After incubation at 95°C for 10 min, the proteins in the supernatant were electrophoresed on a Tricine-SDS-polyacrylamide (4–13%) gel. The protein bands on the gel were stained with the Bio-Rad Silver Stain Plus kit. Proteins in the selected region of the gel were digested with trypsin and identified by tandem mass spectrometry using an Applied Biosystems QStar LC-MS/MS spectrometer (Life Technologies Corp., Carlsbad, CA, USA). The obtained spectrometry information was analyzed with Mascot software (Matrix Science Ltd., London, UK) using the NCBI non-redundant database. The important parameter settings for Mascot analysis were as follows: mass values, monoisotopic; protein mass, unrestricted; peptide mass tolerance, ±0.5 Dalton; fragment mass tolerance, ±0.5 Dalton; and maximal missed cleavages, 2.

### *In Vitro* RdRp Activity Assay

The polymerase activity assay using the RNA molecule embedded within the BaMV replication complex as the template was carried out according to the previous description (Cheng et al., 2001) with slight modification. Briefly, 25 μl P_30_ suspension or S_30Sark_ was included in a final 35 μl reaction solution that also contained 30 mM Tris [pH8.8], 10 mM MgCl_2_, 50 mM NaCl, 20 mM dithiothreitol, 2 mM ATP, 2 mM CTP, 2 mM GTP, 2 μM UTP, 1.5 μl [α-^32^P]UTP (6000Ci/mmol, Perkin Elmer), and 1.5 μl RNase inhibitor. After incubation at 26°C for 3 h, the reaction solution was extracted twice with an equal volume of phenol/chloroform (pH 4.5), and the nucleic acids within were precipitated with ethanol. The radiolabeled RNA products were separated on a 1% agarose gel and analyzed with a phosphoimager (Fujifilm BAS-2500).

### Protein Analysis

Leaves (∼0.1 g) or protoplasts (∼1.5 × 10^4^) were homogenized in buffer that contained 50 mM Tris [pH 7.9], 100 mM KCl, 1 mM EDTA, and 20% glycerol with a handheld polytron in 200 μl cold extraction buffer. The clarified extract after centrifugation at 16200 × g for 10 min was used for protein analysis. The bicinchoninic acid reagent (Pierce) was used to determine the total protein concentration of the samples by using bovine serum albumin as the standard. The relative amounts of the large subunit of ribulose-1,5-bisphosphate carboxylase (L-RuBisCo) in samples were estimated by the intensity of the Coomassie blue-stained protein bands on a 12% SDS-PAGE using Multigauge imaging analysis software (Fujifilm). Viral CPs, GFP, and the HA-tagged protein were detected by western blot analysis using specific CP antisera, GFP antisera, and anti-HA antibodies (Sigma), respectively. The resulting data were processed using Image Station 2000 MM (Kodak).

### RNA Analysis

Leaves (∼0.1 g) were homogenized using 1 ml TriPure Isolation Reagent (Promega, USA). The extracted RNA was dissolved in 50 μl diethyl pyrocarbonate (DEPC)-treated water. The RNA concentration was estimated with a micro-spectrophotometer (NanoDrop Technologies, Wilmington, DE, USA). For genomic and subgenomic RNA analysis, each RNA sample (∼6 μg) was treated with 1.2 M glyoxal and separated on a 1% agarose gel. After transfer and fixation onto a Hybond-N nylon membrane, the samples were hybridized with a DIG-labeled probe complementary to the 3′ UTR of BaMV. The bands hybridized with the probe were visualized by incubating the membrane with anti-digoxigenin-AP Fab fragments and CSPD (Amersham Biosciences, UK). The probe was produced from an *in vitro* transcription reaction that contained 1 μg *Hin*dIII-cleaved pBaHB (Lin et al., 1993), 50 mM DTT, 1 mM ATP, 1 mM CTP, 1 mM GTP, 0.35 mM DIG-UTP, 20 U RNasin, and 40 U SP6 polymerase in 20 μl 1 × SP6 transcription buffer. After incubation at 37°C for 2 h, pBaHB was removed by RNase-free DNase, and the DIG-labeled probe was recovered by ethanol precipitation.

For siRNA analysis, the total RNA extracted from leaves was incubated with 10% PEG-8000 and 1.5 M NaCl on ice for 1 h. The supernatant after centrifugation at 12000 × *g* for 15 min was further incubated with an equal volume of isopropanol and glycogen (5 μg per ml) at - 20°C for at least 2 h. The precipitated RNA after centrifugation was dissolved in DEPC-treated water and electrophoresed on a 15% polyacrylamide-8 M urea gel. After transfer and fixation onto a Zeta-probe blotting membrane (Bio-RAD, USA), the sample was hybridized with a ^32^P-labeled probe that was prepared by an *in vitro* transcription reaction, similar to that for the DIG-labeled probe, except DIG-UTP was replaced with 2 μM UTP and 100 μCi [α-^32^P]UTP (6,000 Ci/mmol). The signal on the membrane was detected with the Fujifilm BAS-2500 phosphorimager.

For semi-quantitative RT-PCR, the total RNA extracted from leaves was primed to synthesize cDNA using an oligo(dT) primer and MMLV high performance reverse transcriptase (Epicenter, USA). A cDNA fragment of NbXRN4, located 179 bp downstream of the fragment that was cloned into pTRV2, was amplified by PCR using primers 5′-GTTTTGCCTGTGGTCAGGTT-3′ and 5′-CTGCCGCCTGAATAAAATGT-3′, while a fragment of b-actin cDNA was amplified using primers 5′-GATGAAGATACTCACAGAAAGA-3′ and 5′-GTGGTTTCATGAATGCCAGCA-3′. PCR was performed under the condition: 5 min at 95°C, 32 cycles of 20 s at 98°C, 15 s at 60°C, and 15 s at 72°C, followed by 5 min at 72°C.

## Results

### BaMV Replication Complex

**FIGURE 1:**
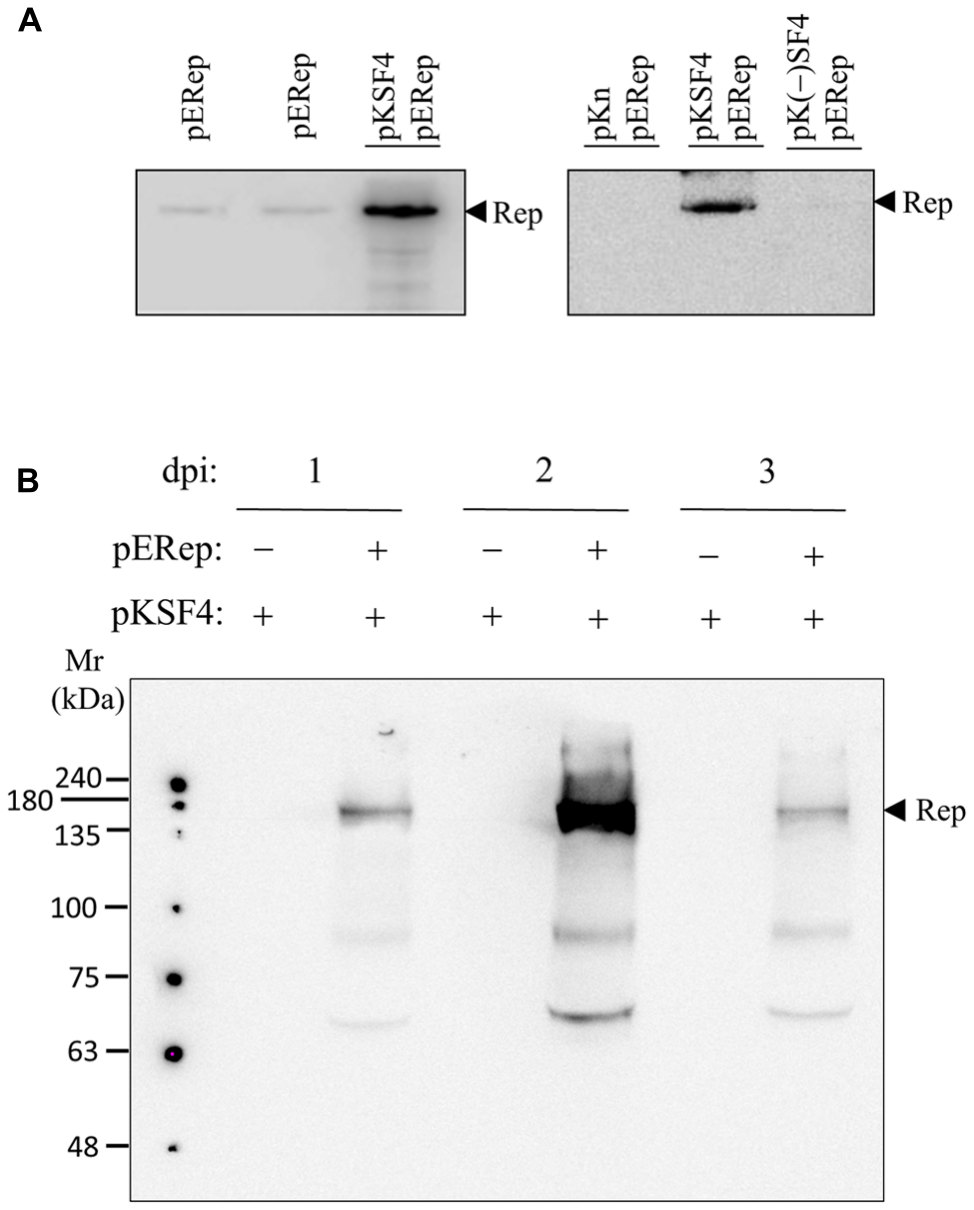
**satBaMV SF4-assisted accumulation of BaMV replication protein.** Leaves of 4-week-old *Nicotiana benthamiana* were agroinfiltrated with the indicated binary plasmids. The P_30_ fraction of the leaves collected on days 2 **(A)** or days 1, 2, and 3 **(B)** after agroinfiltration was isolated, and the presence of BaMV replication protein was examined by western blotting using anti-HA tag antibody. The plasmids and the condition for agroinfiltration are described in Section “Materials and Methods”.

**FIGURE 2:**
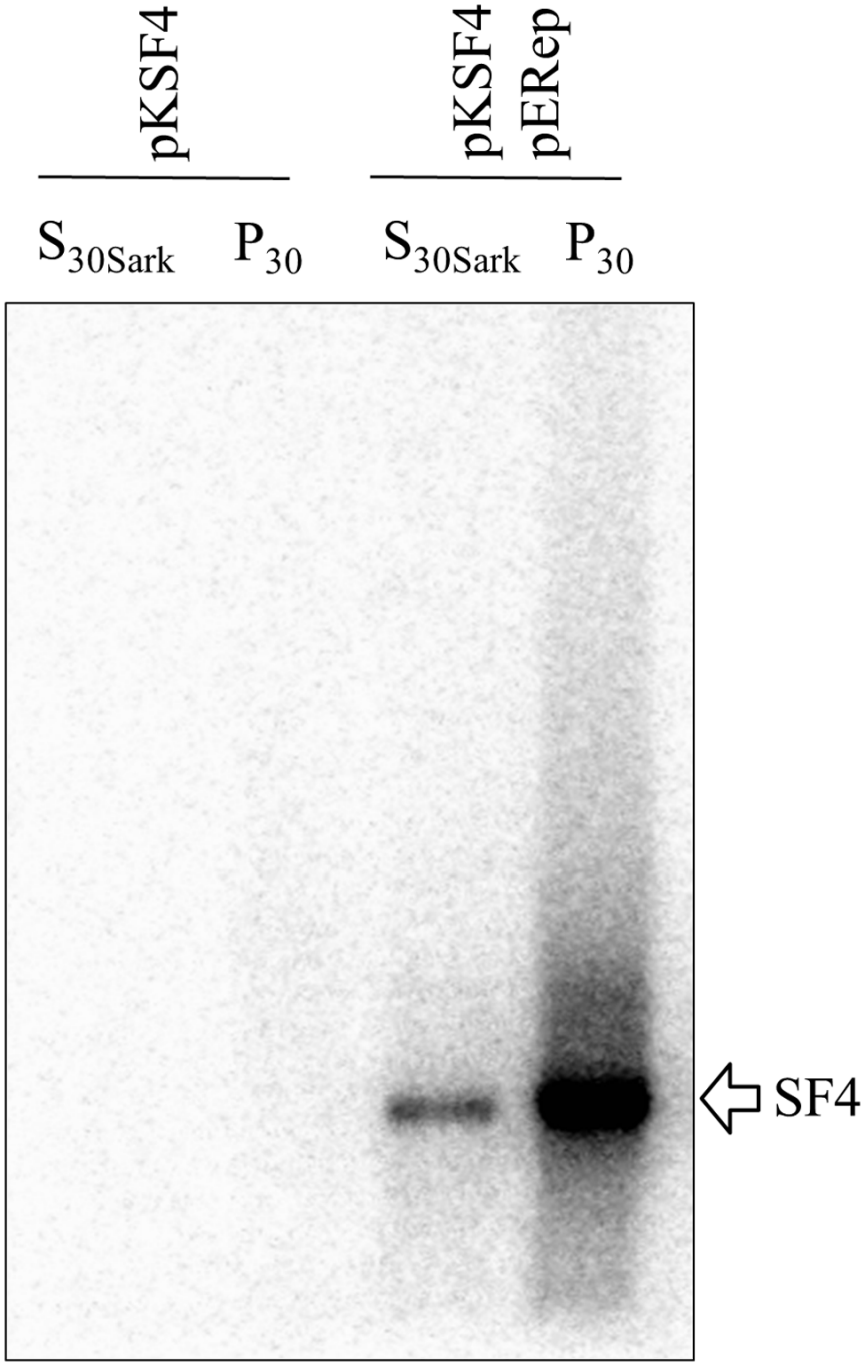
**Exhibition of an endogenous RNA polymerase activity by BaMV replication protein.** P_30_ and S_30Sark_ fractions were prepared from *N. benthamiana* leaves on day 2 after agroinfiltration with the indicated binary plasmids. The reaction condition for the activity assay is described in Section “Materials and Methods”. The radiolabeled RNA products were separated on a 1% agarose gel and visualized with a phosphoimager.

The P_30_ membrane fraction isolated from BaMV-infected leaves has long been known to exhibit an *in vitro* polymerase activity specific for BaMV RNAs, by which the critical *cis*-acting elements on both positive and negative strands of BaMV genome have been determined (Chen et al., 2003, 2005, 2010; Lin et al., 2005). Nonetheless, the BaMV replication protein *per se* could not be detected by western blotting in such a protein preparation, and this limitation has been a hurdle to the characterization of BaMV replication complex in terms of its composition, assembly mechanism, and intracellular localization. In a recent experiment aiming to produce a discernible level of BaMV replication protein in plants, a binary plasmid pERep that harbors the cDNA of BaMV ORF1 in fusion with a HA-coding sequence at the 3′ end was introduced into *N. benthamiana* leaves via *A. tumefaciens* infiltration (Lee et al., 2011). Of the trials, we found that the viral replication protein in P_30_ can be greatly increased if the leaves are co-infiltrated with *A. tumefaciens* that carries pKSF4, an expression vector to produce the SF4 variant of satBaMV (**Figure 1A**). It is noteworthy that co-infiltration with pK(-)SF4, a plasmid for the transcription of the complementary strand of SF4, did not show a similar effect. The accumulation of BaMV replication protein in the infiltrated leaves was monitored as a function of time. The viral protein in P_30_ was discernible on day 1 and reached maximum on day 2 after infiltration (**Figure 1B**). To know whether the viral replication protein in P_30_ is functionally active, an RNA polymerase activity assay using the endogenous RNA embedded in the putative replication complex as the template was performed (**Figure 2**). The P_30_ prepared from leaves co-infiltrated with pERep and pKSF4 was able to generate a RNA product with a size consistent with that of SF4. By contrast, no RNA was produced by the P_30_ from leaves infiltrated with only pKSF4. Based on these results, we propose that the satellite RNA molecule transcribed from pKSF4 acts as a scaffold to promote the correct folding of BaMV replication protein or/and the assembly of the replication complex. As a result, the viral replication protein is ready to replicate satBaMV SF4 and the protein itself is secured from protease degradation due to the complex structure.

### Potential Host Factors

**FIGURE 3:**
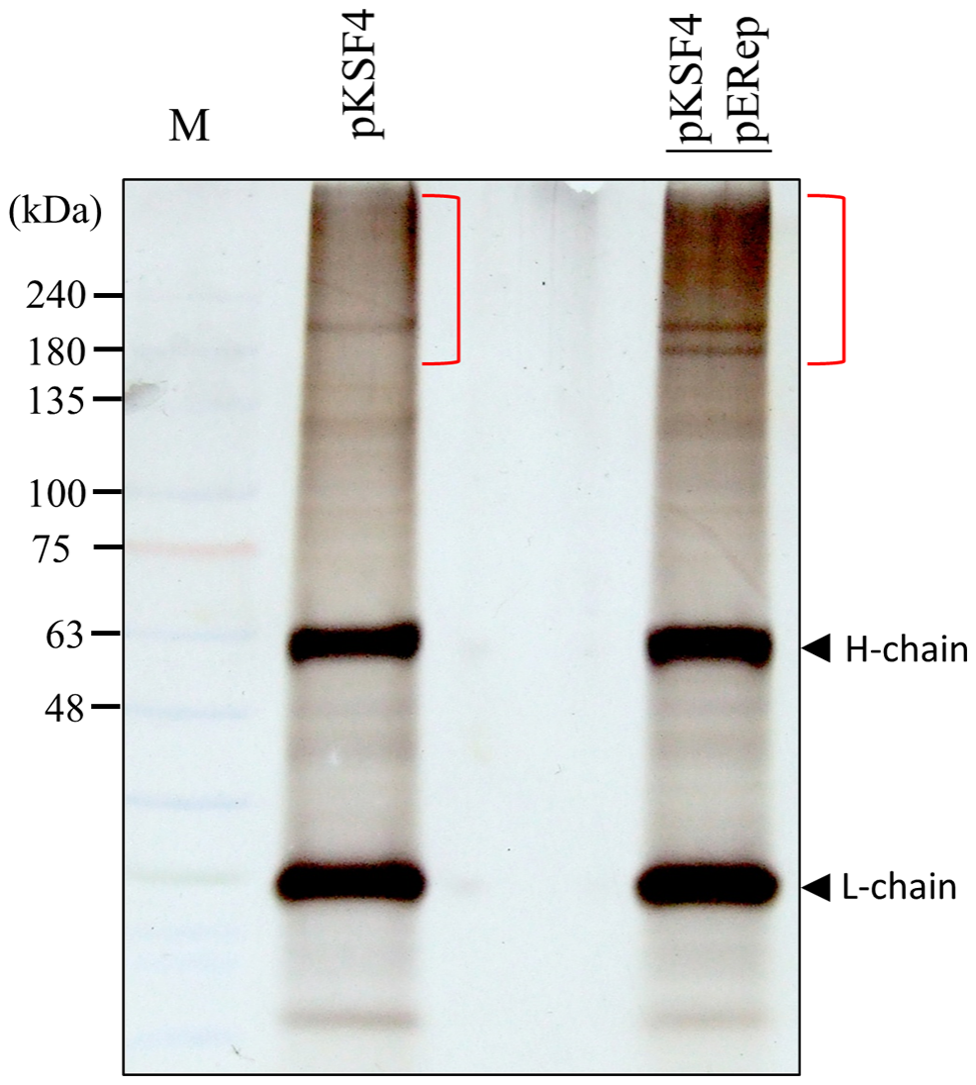
**Analysis of proteins bound to anti-HA agarose beads by SDS-PAGE.** S_30Sark_ was prepared from *N. benthamiana* leaves on day 2 after agroinfiltration with the indicated binary plasmids, and incubated with anti-HA agarose beads. The immuno-precipitate was analyzed by SDS-PAGE. H-chain and L-chain denote the heavy and light chains of the antibody, respectively. The gel region subjected to LC-M/M analysis is indicated with the close bracket.

The activity of a viral replication protein may be modulated by an array of host proteins. Different accessary proteins may be needed at the different stages of the replication process; inversely, some host proteins may be exploited to suppress the polymerase activity of the invading virus. Inspired by the improved production of BaMV replication protein, we set out to look for host proteins in the putative replication complex. First, we tried to solubilize the viral protein in P_30_ with a variety of detergents. The anionic detergent SDS or Sarkosyl at 0.3% (w/v) could release a fraction of the viral protein from P_30_, while non-anionic detergents were barely effective (data not shown). The RNA polymerase activity assay indicated that BaMV replication protein in the Sarkosyl-solubilized solution, S_30Sark_, was still functional in the synthesis of satBaMV (**Figure 2**). Anti-HA agarose beads were added into S_30Sark_ to immunoprecipitate BaMV replication protein. The proteins bound to the beads were analyzed by SDS-PAGE. In comparison with the background control, the sample from leaves agroinfiltrated with pKSF4 and pERep showed some extra protein bands on the gel, particularly in the high molecular weight region (**Figure 3**). To identify the proteins differentially present in the BaMV replication protein-containing sample, the marked regions of the gel, as indicated in **Figure 3**, were sliced and the proteins within were analyzed by LC-MS/MS spectrometry. The plant proteins hit by mass spectrometry in the two samples were compared and those appeared only in the BaMV replication protein-containing sample were chosen for further analysis. They are functionally diverse and could be grouped tentatively into RNA-processing enzymes, disease resistance-associated proteins, development/remodeling-related proteins, and metabolic enzymes (**Table 1**). It is noted that the plausible molecular weights of the selected proteins vary greatly, ranging from ∼34.0 to 161.7 kDa. Presumably, they are in association with macrocomplexes that may consist of proteins, RNAs, lipids, and detergent, and the denaturing condition of the electrophoresis in this study was not strong enough to take the complexes apart completely. In addition, these host proteins are not necessary to be present in the complex for BaMV replication because they were identified from the replicase that assembled on satBaMV. Therefore, the involvement of them in BaMV replication needed to be examined by virtue of the virus-induced gene silencing method.

**Table 1:** Plant proteins differentially present in the BaMV replication protein-enriched fraction.

GI number^1^	Protein	Mascot score
**Nucleic acid processing enzymes:**		
gi| 359490274	DEAD-box ATP dependent RNA helicase 7-like	72
gi| 357113938	ATP-dependent DNA helicase Q-like 2-like	69
gi| 3776009	RNA helicase	67
gi| 242037819	Exoribonuclease (XRN4)	43
**Disease resistance-associated proteins:**		
gi| 356540231	MAP kinase phosphatase-like protein	20
gi| 255563929	Disease resistance protein RGA2	49
gi| 359754963	Ripening-related protein	97
gi| 20522008	Pleiotropic drug resistance like protein	89
**Development/remodeling-related proteins:**		
gi| 30694805	Scarecrow-like protein 5	83
gi| 7576225	ClpA regulatory subunit of Clp protease complex	72
gi| 6715512	Vacuolar H^+^-ATPase B subunit	89
**Metabolic enzymes:**		
gi| 7573308	NADP^+^-dependent isocitrate dehydrogenase	72
gi| 193290730	S-adenosylmethionine synthetase	43
gi| 28268680	Respiratory burst oxidase homolog	69

*^1^The best hit based on the mapped sequences obtained from mass spectrometry. Most of the hit proteins are from plants whose genomes have been sequenced.*

**FIGURE 4:**
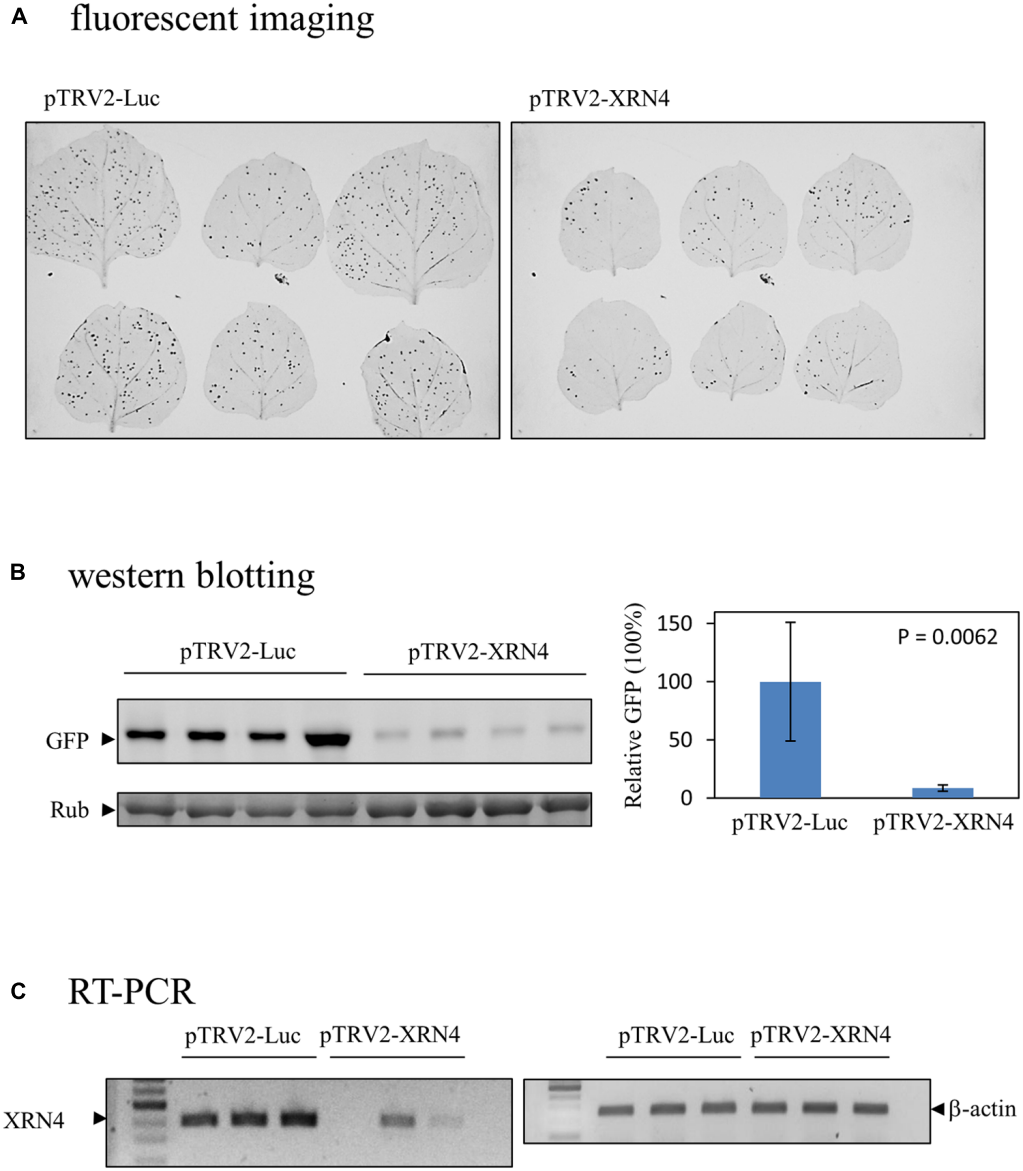
**Reduction of the BaMV-encoded GFP in the NbXRN4-silenced *N. benthamiana*. (A)** Fluorescent images of virus-infected leaves of *N. benthamiana* that had been agroinfiltrated with pTRV1/pTRV2-Luc or pTRV1/pTRV2-XRN4. Two leaves were inoculated with *GFP*-carrying BaMV per independent plant, with a total of three plants in one tested condition. **(B)** The relative expression of GFP due to BaMV infection in leaves agroinfiltrated with pTRV1/pTRV2-Luc or pTRV1/pTRV2-XRN4. The leaves were gathered from four independent plants per tested condition. **(C)** The relative amounts of NbXRN4 and β-actin transcripts in leaves obtained from the plants as described in **(A)**. The RNA extracted from the leaves was converted to cDNA by reverse transcriptase, and the amounts of NbXRN4 and β-actin were examined by PCR using specific primers as described in Section “Materials and Methods”. The *P*-value represents the comparison of groups by Student’s *t*-test (tail = 1, type = 1).

To obtain the cDNAs encoding the identified proteins described above, the expressed sequence tags (ESTs) of *N. benthamiana* in NCBI database were searched using the proteins as queries by the tBLASTn program. The matched ESTs with their ID numbers are listed in **Table 2**. A cDNA fragment of each of the ESTs was amplified by PCR from a leaf cDNA library of *N. benthamiana*. The primers used in the PCR reactions and the sizes of the products are shown in **Table 2**. Each amplified fragment was cloned into pTRV2 vector, and the resulting vector with pTRV1were co-introduced into *N. benthamiana* leaves via agroinfiltration to induce the silence of the target gene. A variety of phenotypic effects could be observed when different genes were silenced. The ClpA regulatory subunit of Clp protease complex-silenced plant grew slowly and showed yellowish leaves. The *S*-adenosylmethionine synthetase-silenced plant showed normal leaves but had underdeveloped buds. Other silenced plants showed negligible changes in their appearances. The silenced plants were then inoculated with *GFP*-carrying BaMV virions, and the development of foci with green fluorescence in the inoculated leaves was scrutinized on day 4 after virus inoculation. The differential GFP imaging in leaves, in comparison with the control, was used to screen potential host factors. Silencing of the gene encoding NbXRN4 significantly decreased GFP expression (**Figure 4A**). The ripening-related protein, *S*-adenosylmethionine synthetase, and the respiratory burst oxidase homolog exerted similar effects as NbXRN4, but at less extent (data not shown). By contrast, silencing of the genes encoding NADP^+^-dependent isocitrate dehydrogenase and MAP kinase phosphatase-like protein appeared to increase GFP expression (data not shown). No apparent difference with respect to GFP expression was noted when the other factors were silenced.

**Table 2:** Sequence number of the EST in *Nicotiana benthamiana* corresponding to the targeted proteins and the primers used in the construction of TRV2 derivatives.

Targeted protein	EST ID^1^	Primers for pTRV2-based construct (5′→3′)^2^	PCR product (bp)
DEAD-box ATP dependent RNA helicase 7-like	CN748199	FP: TTCCGGAATTC*CGATAGTGTCATACCCGCATTTA* RP: TTCCGCTCGAG*CAAGTCCTCAGCAGATACATCAA*	319
	GO608259	FP: TTCCGGAATTC*CATTTGTTTTGCCCATATTAGAGTC* RP: TTCGGCTCGAG*ACTGAAAAGAATTGTTTGAACTTGG*	419
ATP-dependent DNA helicase Q-like 2-like	GO601475	FP: TTCCGGAATTC*AATTCTCAACAAACTCCCTCACA* RP: TTCCGCTCGAG*GTCTATAATCCGGTCGAAAATCA*	459
	CK283590	FP: TTCCGGAATTC*CCAGCATGTGGAAATTGTCTCG* RP: TTCGGCTCGAG*AGCATTCGCAGCGGTAAAGTAG*	403
RNA helicase	CK286108	FP: TTCCGGAATTC*TGATTGTTTATTTAGCATGGGATTT* RP: TTCCGCTCGAG*AAACAGAAGGTTCAATACCCTCTTC*	354
	CK282547	FP: TTCCGGAATTC*GACAGCTTATTAAGATCCTTCAGCA* RP: TTCCGCTCGAG*AGCTAGCAAGAACAACAAACATTCT*	317
Exoribonuclease (XRN4)	EH369301	FP: TTCCGGAATTC*GCTATTGATGGAGTGGCTCCTAGA* RP: TTCGGCTCGAGACCCTCACCAGGAACATTAGCAT	311
MAP kinase phosphatase-like protein	GO612773	FP: TTCCGGAATTC*TTTGTGCACTGCTACCAAGGAGTGT* RP: TTCCGCTCGAG*ATTCTAAGCACAGAACTCGGACT*	212
Disease resistance protein RGA2	CK290351	FP: TTCCGGAATTC*GCTGTAGGTTTATCTTTTGATGAT* RP: TTCCGCTCGAGGATTCTCCATTCCTTCTATTCTT	467
Ripening-related protein	ES886818	FP: TTCCGGAATTC*CACTTTCTGTTCACAACTTATTGC* RP: TTCGGCTCGAG*CTCATACATAATTGTCCAAGTAGTC*	410
Pleiotropic drug resistance like protein	CK291986	FP: TTCCGGAATTC*ATGTTGTATACTCCGTTGACATGC* RP: TTCGGCTCGAG*GGGTCCAAGCTCAACAAGTTCC*	404
Scarecrow-like protein 5	CN744167	FP: TTCCGGAATTC*TCCAGAAGTCACTAAGGCTATGC* RP: TTCGGCTCGAG*GAAAACCTGCCATCATGAACCTG*	416
ClpA regulatory subunit of Clp protease complex	GO604713	FP: TTCCGGAATTC*TGATGAAGCTGGTTCTCGTGTTC* RP: TTCGGCTCGAG*CGTCGAATGGCACGACTAATGG*	411
vacuolar H+-ATPase B subunit	GO601484	FP: TTCCGGAATTC*TTCGGTTGGGAGATGGAACTACT* RP: TTCCGCTCGAG*ATAGCAGCAAAGACAATGGCAAA*	493
NADP^+^-dependent isocitrate dehydrogenase	EX534040	FP: TTCCGGAATTC*CTTATCTTTCCCTTTGTGGAGTT* RP: TTCCGCTCGAG*ATCTTTTCATCCTTCCCTTCTGGTA*	407
S-adenosylmethionine synthetase	CK290599	FP: TTCCGGAATTC*ATTTACCTCCGAGTCTGTGAACG* RP: TTCGGCTCGAG*CTGTGGCATACCCGAACATGTG*	380
Respiratory burst oxidase homolog	CK292677	FP: TTCCGGAATTC*ACCATCATTCGGACACAGAGATAAT* RP: TTCGGCTCGAG*AAGCATTTCGAACAAGTGAAGATCC*	330

*^1^The entity of expressed sequence tag (EST) in *N. benthamiana* was obtained through search using tBASLTn.*

*^2^Nucleotide sequences of forward primer (FP) and reverse primer (RP) used in the PCR to obtain the cDNA fragments for the construction of pTRV2 derivatives. The underlined sequences are engineered restriction sites.*

### Decrease of BaMV in *N. benthamiana* by NbXRN4 Silencing

Among the screened proteins, NbXRN4 had the most prominent effect on the development of green fluorescent foci. The protein extract of the virus-inoculated leaves was analyzed by western blotting using anti-GFP antiserum to sustain the difference in fluorescent images. The average amount of GFP, normalized against L-RubisCo, in leaves of the NbXRN4-silenced plants was about one tenth of that in the control plants (**Figure 4B**), consisting with the fluorescent imaging of leaves. RT-PCR was performed to confirm the silencing efficiency of NbXRN4 according to the description in Section “Materials and Methods”. The amplified amounts of the cDNA fragment of NbXRN4 in the plants agroinfiltrated with pTRV1/pTRV2-XRN4 were considerably lower than that in the control plants, as β-actin was used as the internal control (**Figure 4C**). These results indicate that downregulation of NbXRN4 actually decreased the accumulation of GFP, a translation product of the ∼1.8-kb subgenomic RNA of *GFP*-carrying BaMV.

**FIGURE 5:**
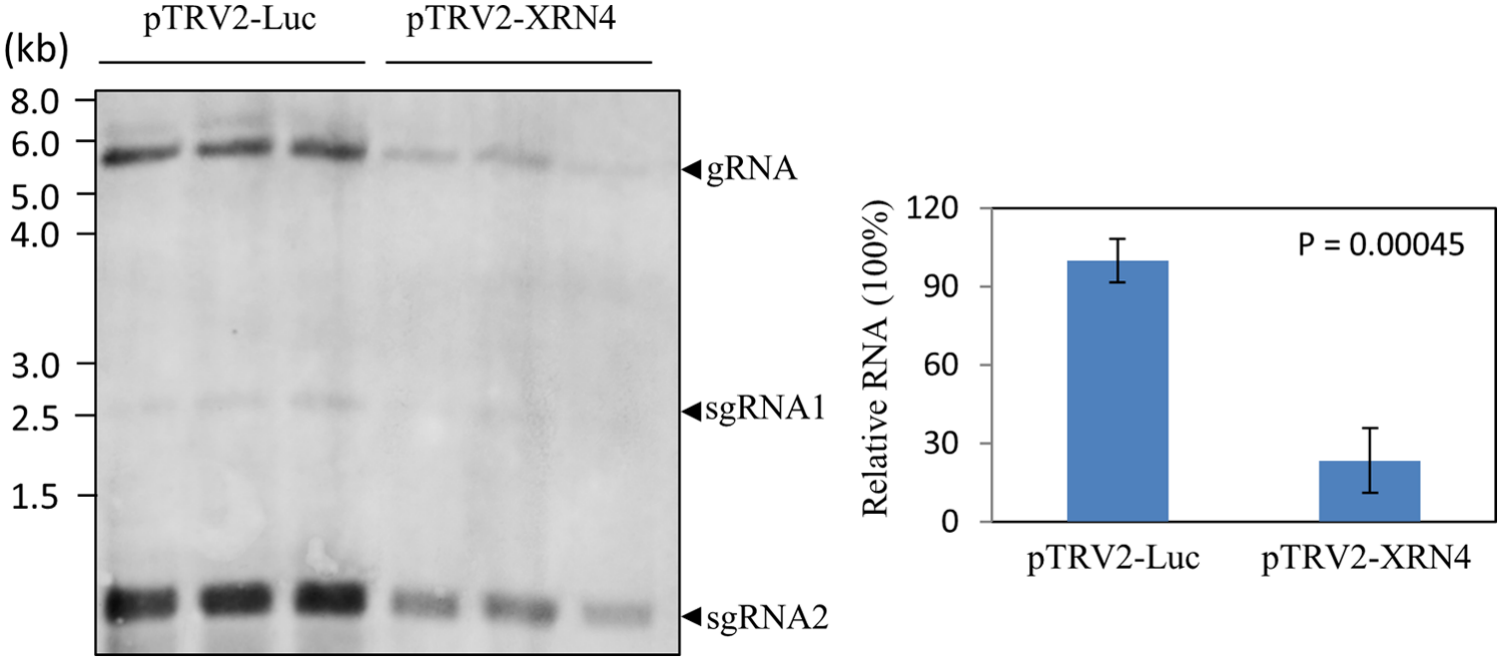
**Reduction of the positive-sense RNAs of BaMV in the NbXRN4-silenced plants.** RNA was extracted from BaMV-infected leaves of *N. benthamiana* that had been agroinfiltrated with pTRV1/pTRV2-Luc or pTRV1/pTRV2-XRN4. Leaves were collected from three independent plants per tested condition. RNA molecules separated on an agarose gel were hybridized with a probe complementary to the 3′ UTR of BaMV. The viral genomic (gRNA) and two major subgenomic (sgRNA) RNAs are indicated. The relative accumulations of BaMV genomic RNA were compared quantitatively in the right panel. The *P-*value represents the comparison of groups by Student’s *t*-test (tail = 1, type = 1).

To know whether NbXRN4 exerted a differential effect on accumulations of the viral genomic and subgenomic RNAs, the NbXRN4-silenced plants were inoculated with BaMV virions and the total RNA extracted from the leaves 4 days post-inoculation was analyzed by northern blotting using a probe complementary to the 3′ UTR of BaMV (**Figure 5**). Accumulations of both genomic and subgenomic RNAs in the NbXRN4-silenced plants dropped to about a quarter of that in the control plants, indicating that silencing of NbXRN4 could either have an inhibitory effect on the viral replication or stimulate the turnover of the viral RNAs. Moreover, the decrease in GFP expression should result from the general reduction in the viral RNAs.

**FIGURE 6:**
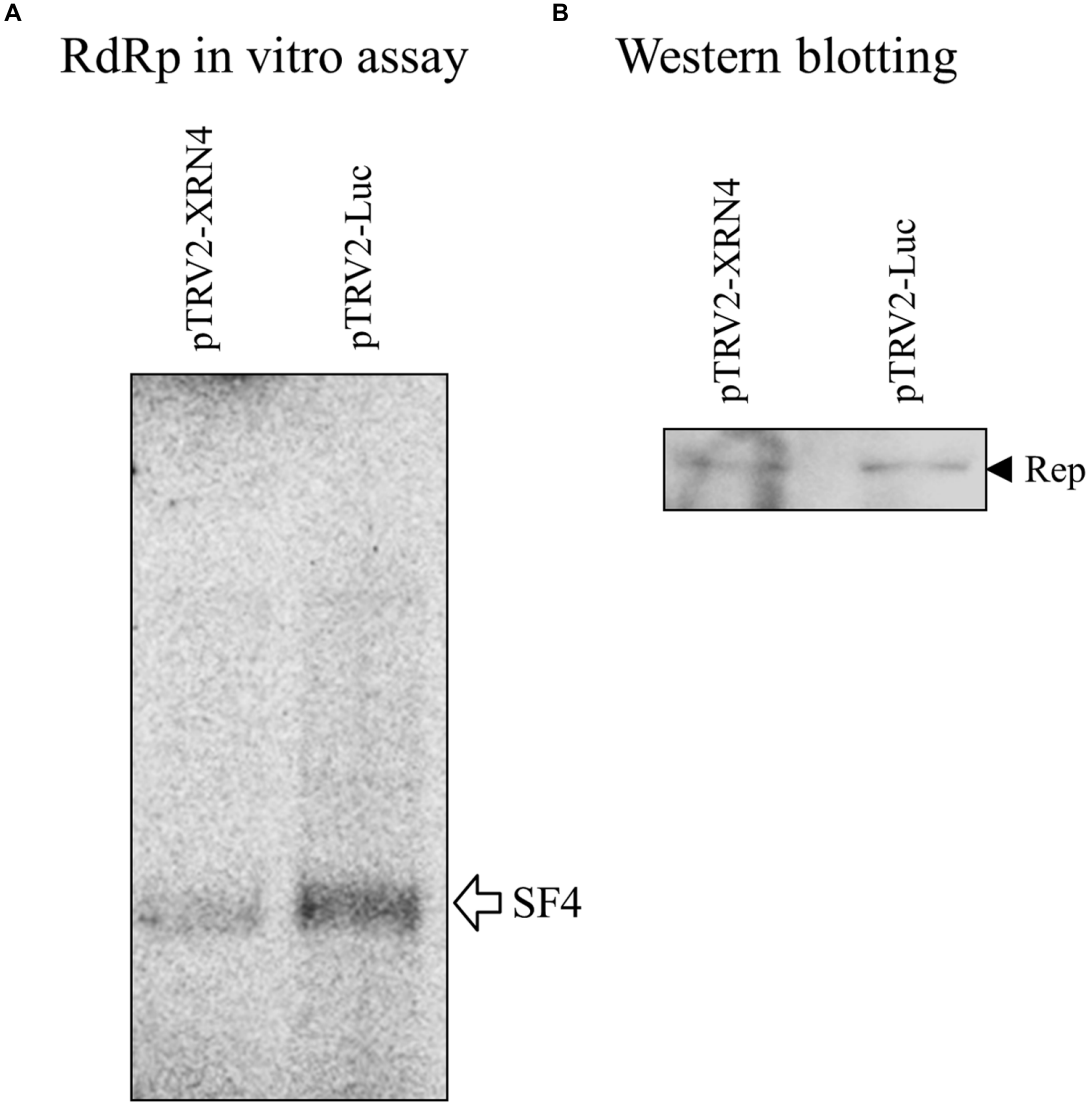
**Suppression of satBaMV synthesis by NbXRN4 silencing.** *N. benthamiana* was agroinfiltrated with pTRV1/pTRV2-Luc or pTRV1/pTRV2-XRN4. After 3 weeks, the leaves of the silencing plants were co-infiltrated with pERep and pKSF4. The P30 fraction was isolated on day 2 after infiltration, and **(A)** the *in vitro* RdRp activity for satBaMV synthesis was assayed as described in Section “Materials and Methods”. **(B)** The relatively amounts of the BaMV replication protein used in the activity assays are indicated by western blotting.

Since NbXRN4 was initially identified from the putative replication complex based on satBaMV, it was interesting to know whether this host protein exerts a similar function regarding the accumulation of both BaMV and satBaMV. Leaves of the NbXRN4-silenced plant were co-infiltrated with pERep and pKSF4, and the P30 fraction was isolated 2 days post infiltration. Subsequently, the *in vitro* RdRp activity of P30 for satBaMV synthesis was assayed. Downregulation of NbXRN4 significantly decreased the yield of satBaMV (**Figure 6**), suggesting that NbXRN4 has a positive effect on the accumulation of genomic RNA as well as satellite RNA.

**FIGURE 7:**
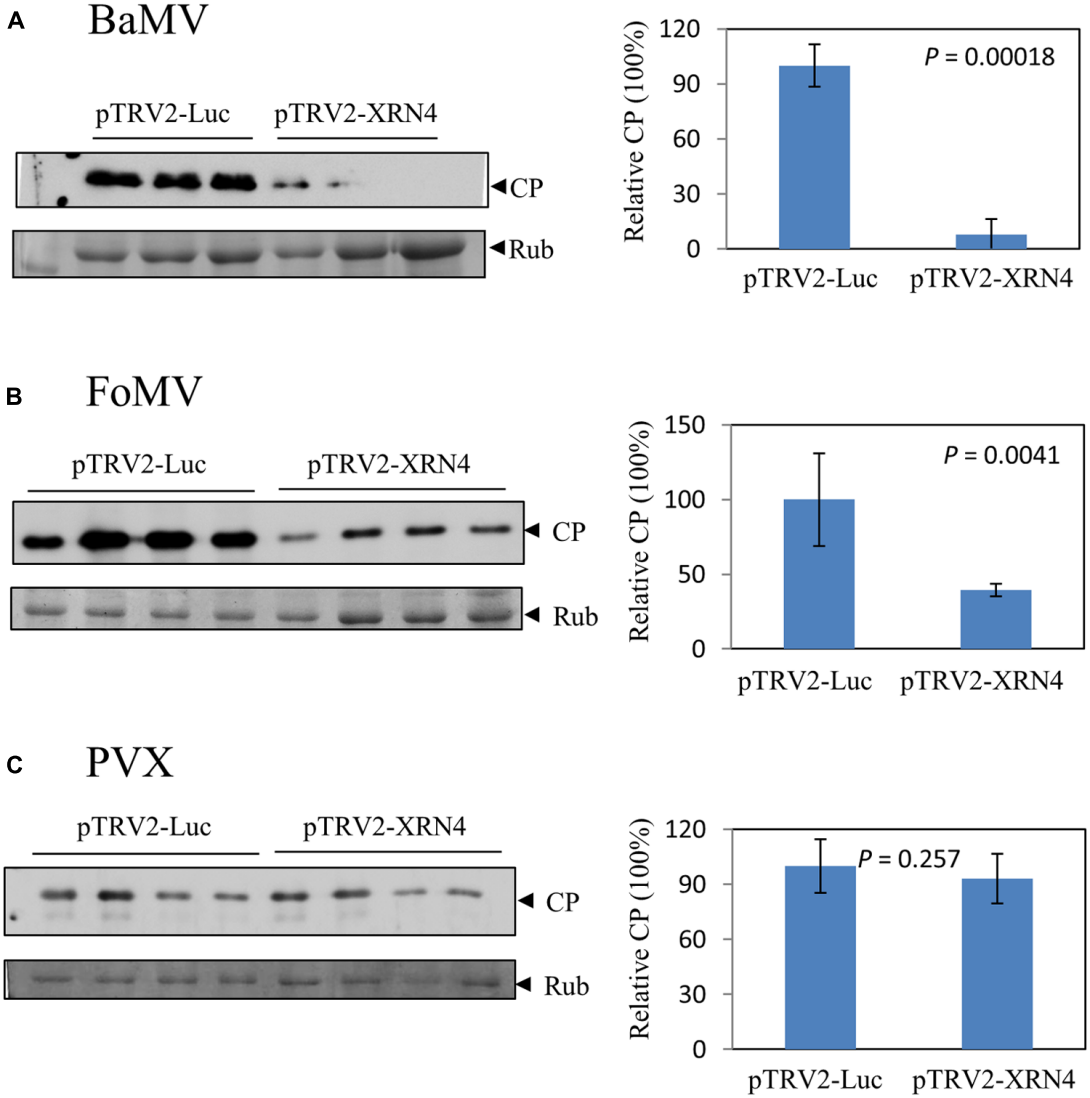
**Differential effects of NbXRN4 on the accumulation of various potexviral CP.** The NbXRN4-silenced *N. benthamiana* was inoculated with *Bamboo mosaic virus* **(A)**, *Foxtail mosaic virus* **(B)**, or *Potato virus X* **(C)**. The relative expression of the viral coat protein (CP) was analyzed by western blotting 2 days post-inoculation. The sample in each lane was from an individual plantlet. *P*-values represent comparisons of groups by Student’s *t*-test (tail = 1, type = 1).

The genus *Potexvirus* consists of many economically important pathogens such as the type species *Potato virus X* (PVX) and *Foxtail mosaic virus* (FoMV). To test whether the effect exerted by NbXRN4 is common to other potexviruses, the NbXRN4-silenced plants were also inoculated with virions of FoMV and PVX. Accumulations of the viral CP in the inoculated leaves were assayed on day 4 after inoculation. The average amount of the accumulated BaMV CP in the NbXRN4-silenced plants was no more than 10% of that in the control plants (**Figure 7A**). As for FoMV, a 60% drop in CP accumulation was observed in the NbXRN4-silenced plants (**Figure 7B**). By contrast, there was no significant effect on the accumulation of PVX CP (**Figure 7C**). BaMV replication protein shares 58 and 43% identities in amino acid sequence with that of FoMV and PVX, respectively. The silencing effect of NbXRN4 appears to relate to the closeness between replication proteins of BaMV and other potexviruses.

### Increase of BaMV by NbXRN4 Overexpression

A couple of mRNA transcripts that may contain the coding region of NbXRN4 are available in the *N. benthamiana*_transcriptome databases, Sydney University, with the ID numbers such as Nbv5tr6403855 and Nbv5tr384653. Further analysis using the BLASTx program against non-redundant protein sequences revealed that the protein encoded by those transcripts has 98% identity over the total 982 amino acid residues with an annotated exonuclease (XP_009765212) of *N. sylvester*. Since the prediction of XP_009765212 was based on the sequenced genome of *N. Sylvester*, the *N. benthamiana* transcripts obtained from the databases of Sydney University are believed to be able to direct the synthesis of a complete NbXRN4. A pair of primers was thus designed to clone the coding region of NbXRN4 from a *N. benthamiana* leaf cDNA library, and the clone was used to substitute for the β-glucuronidase gene in plasmid pBI221.

**FIGURE 8:**
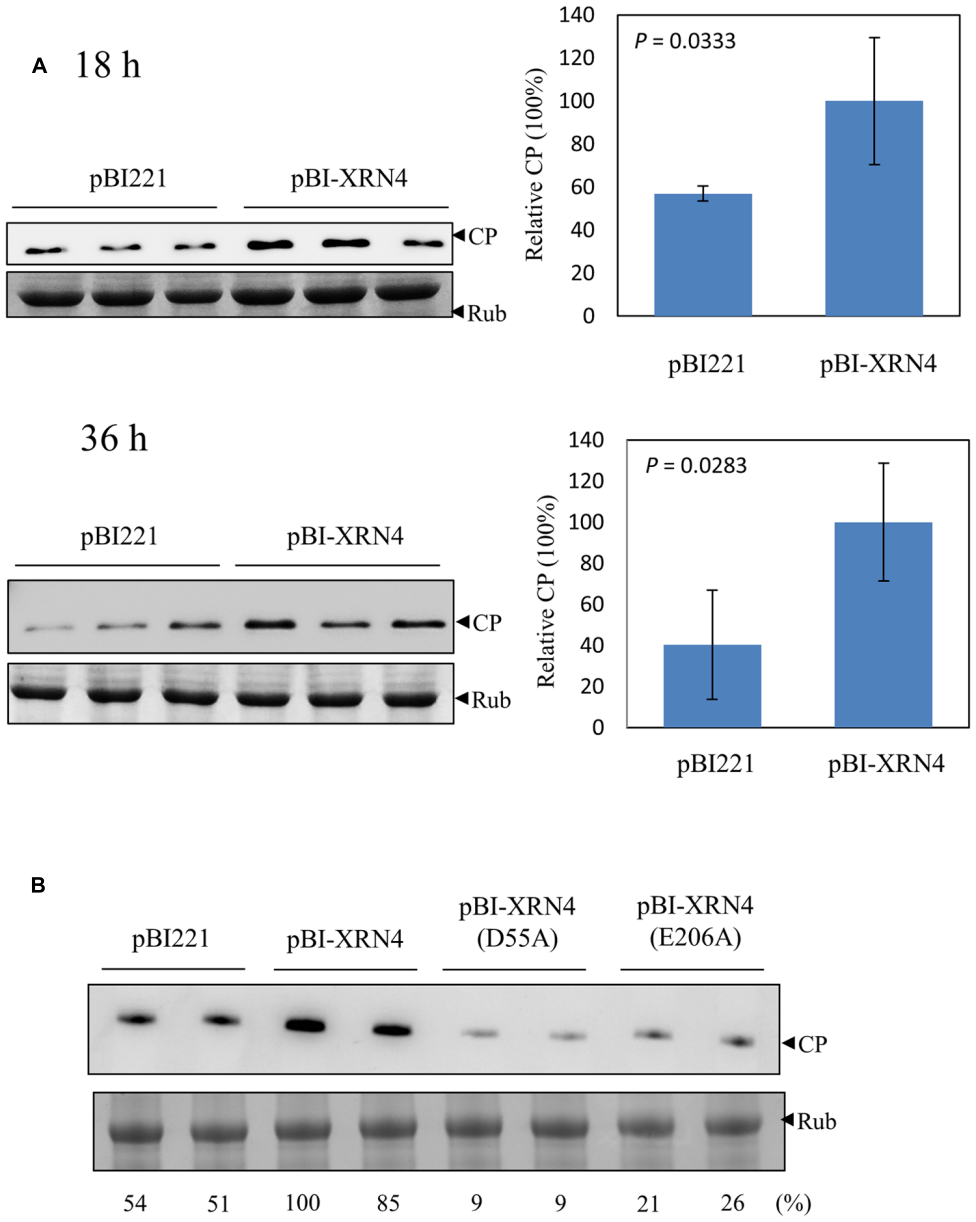
**Increase of BaMV CP in the NbXRN4-overexpressed protoplasts. (A)** *N. benthamiana* protoplasts (1 × 10^5^) were co-transfected with 1 μg pCBG plus 3 μg pBI221, or pBI-XRN4. The accumulation of BaMV CP was analyzed by western blotting 18 and 36 h post-transfection. **(B)** The accumulation of BaMV CP in protoplasts transfected with the indicated plasmid (3 μg) plus pCBG (1 μg) was analyzed 18 h post-transfection. pBI-XRN4(D55A) and pBI-XRN4(E206A) were used to produce mutant NbXRN4 in which the active-site residues Asp55 and Glu206, respectively, were replaced with alanine. The sample in each lane was from an individual plantlet. *P*-values represent comparisons of groups by Student’s *t* test (tail = 1, type = 1).

Silencing of NbXRN4 decreased the accumulations of BaMV RNAs and the virus-encoded proteins; thus, it was important to find out whether overexpression of this specific exonuclease would increase BaMV accumulation. *N. benthamiana* protoplasts were co-transfected with pCBG and the transient protein expression vector pBI221, or pBI-XRN4. The accumulation of BaMV CP was monitored at 18 and 36 h post-transfection. The protoplasts co-transfected with pBI221 accumulated less BaMV CP than those with pBI-XRN4, by 40 and 60% drops at 16 and 36 h, respectively (**Figure 8A**). This data demonstrated a positive effect of NbXRN4 on BaMV accumulation. Because this effect was manifested in protoplasts, it should be regardless of a cell-to-cell movement-related mechanism.

NbXRN4 contains 982 amino acid residues with an N-terminal 5′→3′ exonuclease domain conserved in members of XRN_N family. The active site residues are highly conserved across XRN1s, the main cytoplasmic RNase associated with 5′→3′ mRNA decay in animals and fungi. XRN4 is the functional homolog of XRN1 in plants (Kastenmayer and Green, 2000). The structure of *Drosophila* XRN1 (PACMAN) shows two catalytically essential Mg^2+^ ions coordinated by the active-site residues Asp35, Asp86, Glu177, Asp205, Asp207, and Asp288 (Nagarajan et al., 2013). To know whether the catalytic function of NbXRN4 is critical for promoting the accumulation of BaMV, Asp55 and Glu206 of the protein (corresponding to Asp35 and Glu177 of PACMAN, respectively) were targeted for alanine mutagenesis. The mutated pBI-XRN4 and pCBG were introduced into protoplasts to examine the mutational effects. Neither of the mutant NbXRN4s was able to promote the accumulation of BaMV CP (**Figure 8B**), indicating a critical role of the RNA hydrolysis function in this regard.

### Irrelevance Between the Function of NbXRN4 in BaMV Accumulation and the RNAi Mechanism

**FIGURE 9:**
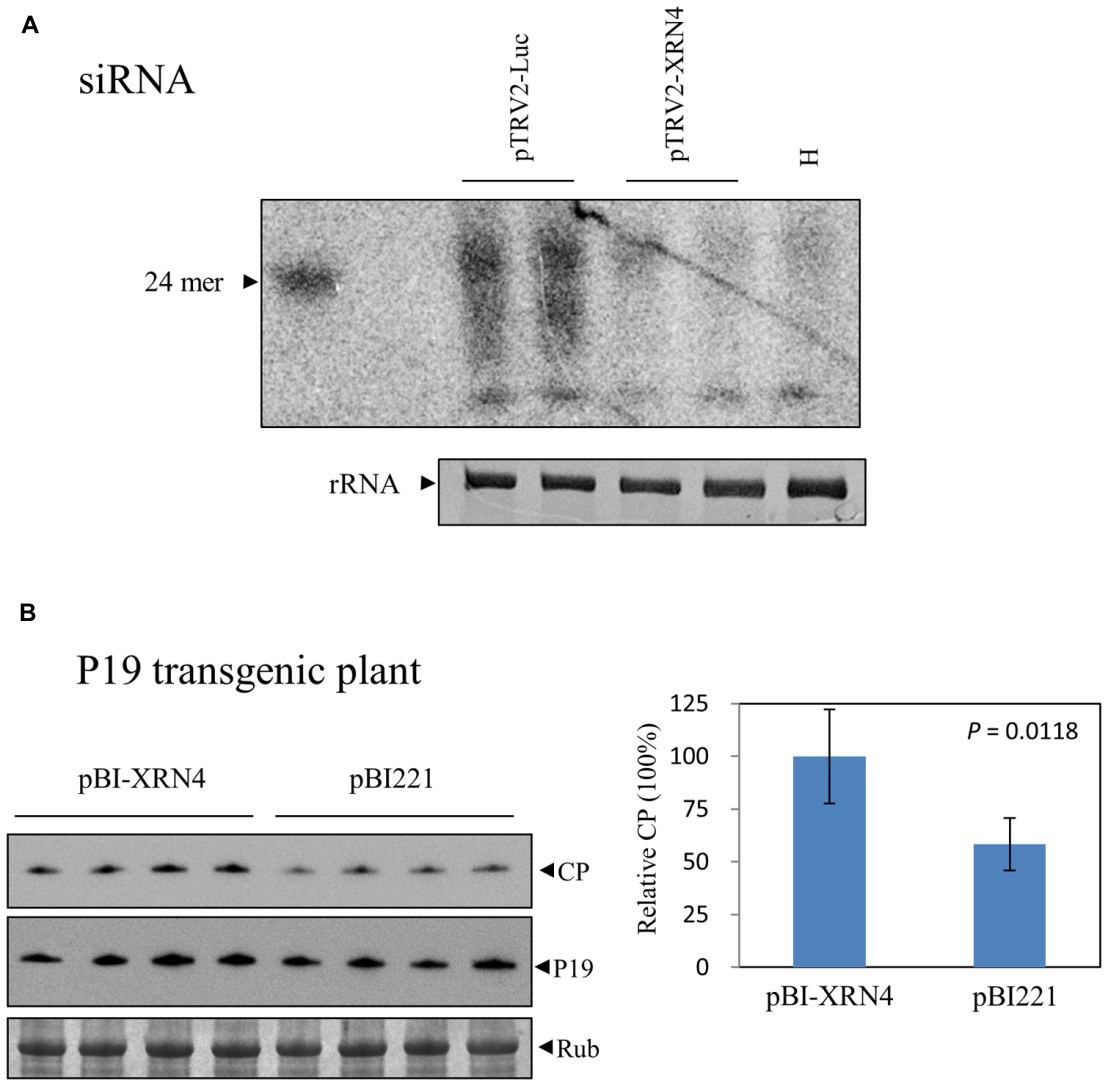
**Irrelevance between the increase of BaMV by NbXRN4 and the RNAi mechanism. (A)** BaMV-specific siRNAs in *N. benthamiana* plants that had been agroinfiltrated with TRV1/TRV2-Luc or TRV1/TRV2-XRN4. RNAs in the plants was isolated by a protocol for selective enrichment of small RNAs on day 4 after BaMV inoculation and analyzed by northern blotting using a probe complementary to the 3′ UTR of BaMV. H denotes the plant without virus inoculation. **(B)** Protoplasts from P19 transgenic *N. benthamiana* were co-transfected with 1 μg pCBG plus 3 μg pBI221, or pBI-XRN4. The accumulations of BaMV CP and P19 were analyzed by western blotting at 18 h after transfection. The sample in each lane was from an individual plantlet. The *P*-value represents the comparison of groups by Student’s *t*-test (tail = 1, type = 1).

Loss of XRN4 in *Arabidopsis* increased siRNA-mediated mRNA decay (Gazzani et al., 2004). This effect has been attributed to the survival of uncapped RNAs in *xrn4* mutants, leading to the formation of double-stranded RNA precursors used for siRNA biogenesis. Mutations of XRN4 also led to the over-accumulation of miRNA-generated cleavage products (Souret et al., 2004). It was thus interesting to investigate whether the decrease in BaMV RNA accumulation in the NbXRN4-silenced plants results from an enhanced RNA degradation via an RNAi-related mechanism. The accumulation of siRNAs derived from BaMV in the virus-infected plants that had been infiltrated with TRV1/TRV2-NbXRN4 or TRV1/TRV2-Luc was examined by northern blotting assay. If an RNAi-related mechanism plays a major role in decreasing the accumulated level of BaMV RNAs, more BaMV siRNAs in the NbXRN4-silenced plants may be expected. The result showed a much less amount of BaMV siRNAs in the NbXRN4-silenced plants than that in the control (**Figure 9A**), in agreement with the accumulated levels of BaMV RNAs in the previous experiment (**Figure 5**), suggesting that the decrease in BaMV RNAs in the NbXRN4-silenced plants should not result from an increased degradation of the viral RNAs via RNAi mechanisms. Overall, more BaMV accumulation due to the presence of NbXRN4 will lead to more BaMV siRNAs generated by the RNAi mechanism.

To further investigate the involvement of RNAi mechanisms in the stimulating function of NbXRN4 in BaMV accumulation, we tested the effect of NbXRN4 in protoplasts derived from a P19 transgenic *N. benthamiana* line. This transgenic line is able to produce P19 (**Figure 9B**) and sustains an enhanced expression of the recombinant VP1 of *Foot-and-mouth disease virus* (unpublished data). In addition, overexpression of P19 in the cell suspension culture of *N. benthamiana* leaves greatly enhanced the production of BaMV chimeric virus particles (Muthamilselvan et al., 2015). If an RNAi-related mechanism plays a major role, the stimulating effect of NbXRN4 should be abolished in the presence of the RNAi suppressor P19. Overexpression of NbXRN4 in the P19 transgenic protoplasts was still able to increase the accumulation of BaMV CP (**Figure 9B**), precluding the involvement of an RNAi-related mechanism in the stimulating function of NbXRN4.

## Discussion

A number of *N. benthamiana* proteins involved in BaMV replication or movement have been identified by approaches using techniques such as cDNA-ALFP and UV-induced crosslinking. In this study, a protocol was established to isolate the BaMV replication protein-enriched fraction, from which a handful of selectively representing proteins were found. Some of them are able to modulate the accumulation of BaMV according to the results of subsequent screen. Elucidation of the functions of those proteins will broaden our knowledges regarding the interplay between BaMV and its plant hosts.

XRN_N family is typically represented by one cytoplasmic enzyme (XRN1/PACMAN or XRN4) and one or more nuclear enzymes (XRN2/RAT1 and XRN3). XRN1 in yeast is the main cytoplasmic RNase associated with 5′→3′ mRNA decay (Garneau et al., 2007; Geisler and Coller, 2012). One of the mRNA turnover pathways starts from removal of the 5′ cap by the enzymes DCP1 and -2, and the uncapped RNA is subsequently degraded by XRN1. In the nucleus, XRN2/RAT1 functions in the processing of rRNAs and small nucleolar RNAs (Petfalski et al., 1998). XRN2 also has an important role in transcription termination by RNA polymerase II (Kim et al., 2004; West et al., 2004). Three XRNs have been identified from *Arabidopsis thaliana*, named AtXRN2, AtXRN3, and AtXRN4. The first two enzymes are targeted to the nucleus, while the cytosolic AtXRN4, the functional homolog of XRN1, is responsible for the decay of uncapped mRNA and miRNA-guided mRNA cleavage products (Kastenmayer and Green, 2000; Souret et al., 2004). In addition, AtXRN4 has been shown to act as an endogenous suppressor of posttranscriptional gene silencing (Gazzani et al., 2004; Souret et al., 2004).

The involvement of XRN1 or XRN4 in viral infections has been reported. Production of a small subgenomic flavivirus RNA, which is important for the pathogenicity of *Yellow fever virus*, from the incomplete degradation of the viral genome requires the 5′→3′ exonuclease activity of XRN1 (Silva et al., 2010). Silencing of NbXRN4 promoted the accumulation of tombusvirus RNAs and enhanced the viral RNA recombination (Jaag and Nagy, 2009), while overexpression of AtXrn4 in *N. benthamiana* accelerated the degradation of the viral RNAs (Cheng et al., 2007). Similarly, silencing of NbXRN4 facilitated both local and systemic infection of *Tobacco mosaic virus* (TMV) in *N. benthamiana* (Peng et al., 2011). To tombusvirus and TMV infection, NbXRN4 seems to act as an antiviral agent. However, an enhancer role of NbXRN4 in BaMV replication was observed in this study. Apparently, diverse biology functions of XRN1 or XRN4 are displayed in relation to virus accumulation by the same 5′→3′ exonuclease activity.

Although XRN1 or XRN4 is generally considered a cytosolic exonuclease, cytoplasm is not necessarily the place where it exerts its control over virus accumulation. More likely, the exonuclease may be recruited into the viral replication complexes by different viruses at specific stages so that it can perform distinctly biological functions. In addition, the silencing suppressor function of cytoplasmic XRN4 should not play a determinant role in the scenario of controlling virus accumulations, because a universal effect on different viruses would otherwise be expected. This notion is also supported by the observation that NbXRN4 was still able to increase BaMV accumulation when the universal silencing suppressor P19 was present.

The effect of NbXRN4 on BaMV accumulation relies on the exonuclease activity of the enzyme; however, the detailed mechanism underlying this effect is yet unknown. Aberrant genomic and subgenomic RNAs without the 5′ cap structure may be generated during the replication/transcription process of BaMV. Presumably, NbXRN4 could help to remove those defective RNAs, which otherwise may interfere with the replication process by competing with the normal RNAs to the viral replication machinery. NbXRN4 may also play a role in editing the 5′ end of the nascent viral RNAs before the RNAs are modified with the 5′ cap structure. Biochemical characterizations of NbXRN4 are needed to clarify such speculative mechanisms in the future.

## Conclusion

Several *N. benthamiana* proteins that may regulate the replication of BaMV were identified from the putative viral replication complex in this study. They include cytoplasmic 5′→3′ exoribonuclease (NbXRN4), *S*-adenosylmethionine synthetase, a ripening-related protein, a respiratory burst oxidase homolog, a MAP kinase phosphatase-like protein, and NADP^+^-dependent isocitrate dehydrogenase. The major function of NbXRN4 in cytoplasm is to degrade uncapped mRNA and miRNA-guided mRNA cleavage products. However, it may exhibit distinct functions in response to the infection of different viruses. NbXRN4 attenuates the accumulation of TBSV and TMV by facilitating the degradation of the viral RNAs. By contrast, NbXRN4 increases the accumulated level of BaMV. The replication efficiency of BaMV may be improved by the exoribonuclease activity of NbXRN4.

## Author Contributions

C-CL, coordinates the experiments performed by the other authors, performed RdRp activity assay and protoplast transfection. T-LL prepared the viral replication protein-enriched fraction and performed the identification of proteins selectively present in the fraction. J-WL prepared the target gene-silenced plants and assayed the viral accumulation in the plants, carried out western and northern blotting assays. Y-TH prepared the target gene-silenced plants. Y-TH performed plasmid construction. Y-HH prepared P19 transgenic plant and being involved in discussion. MM planed the experiments and prepared the manuscript.

## Conflict of Interest Statement

The authors declare that the research was conducted in the absence of any commercial or financial relationships that could be construed as a potential conflict of interest.
